# Meristem size contributes to the robustness of phyllotaxis in *Arabidopsis*

**DOI:** 10.1093/jxb/eru482

**Published:** 2014-12-11

**Authors:** Benoit Landrein, Yassin Refahi, Fabrice Besnard, Nathan Hervieux, Vincent Mirabet, Arezki Boudaoud, Teva Vernoux, Olivier Hamant

**Affiliations:** ^1^Laboratoire de Reproduction de développement des plantes, INRA, CNRS, ENS Lyon, UCB Lyon 1, Université de Lyon, 46 Allée d’Italie, 69364 Lyon, Cedex 07, France; ^2^Laboratoire Joliot-Curie, Laboratoire de Physique, CNRS, ENS Lyon, UCB Lyon 1, Université de Lyon, 46 Allée d’Italie, 69364 Lyon, Cedex 07, France; ^3^Sainsbury Laboratory, University of Cambridge, Cambridge CB2 1NN, UK; ^4^Institut Universitaire de France, 103, boulevard Saint-Michel, 75005 Paris, France

**Keywords:** Meristem, morphometry, patterning, phyllotaxis, plastochrone, robustness.

## Abstract

Using the plant model *Arabidopsis*, the relationship between day length, the size of the shoot apical meristem, and the robustness of phyllotactic patterns were analysed. First, it was found that reducing day length leads to an increased meristem size and an increased number of alterations in the final positions of organs along the stem. Most of the phyllotactic defects could be related to an altered tempo of organ emergence, while not affecting the spatial positions of organ initiations at the meristem. A correlation was also found between meristem size and the robustness of phyllotaxis in two accessions (Col-0 and WS-4) and a mutant (*clasp-1*), independent of growth conditions. A reduced meristem size in *clasp-1* was even associated with an increased robustness of the phyllotactic pattern, beyond what is observed in the wild type. Interestingly it was also possible to modulate the robustness of phyllotaxis in these different genotypes by changing day length. To conclude, it is shown first that robustness of the phyllotactic pattern is not maximal in the wild type, suggesting that, beyond its apparent stereotypical order, the robustness of phyllotaxis is regulated. Secondly, a role for day length in the robustness of the phyllotaxis was also identified, thus providing a new example of a link between patterning and environment in plants. Thirdly, the experimental results validate previous model predictions suggesting a contribution of meristem size in the robustness of phyllotaxis via the coupling between the temporal sequence and spatial pattern of organ initiations.

## Highlight

Phyllotaxis describes the regular position of leaves and flowers along plant stems. It is demonstrated that errors in this pattern can be related to meristem size and day length.

## Introduction

How molecular pathways and interactions lead to reproducible shapes is at the centre of today’s developmental biology. Conversely, the geometry of a multicellular shape can impact on cell activity, also contributing to the robustness of patterns in development. Here this feedback was investigated using the shoot apical meristem (SAM) of *Arabidopsis* as a model system. SAMs are highly organized groups of dividing cells whose activity is responsible for the emergence of all the aerial organs of plants (Bowman and Eshed, 2000). In most angiosperms, successive organs are initiated at a relative angle, called the divergence angle (α), of ~137° at the meristem, and such regularity has fascinated botanists and mathematicians since antiquity (Adler *et al.*, 1997). There is evidence today that this regularity is controlled by the distribution of the plant hormone auxin within the meristem. Auxin has been shown to play a key role in organ initiation at the meristem, notably by defining the position of subsequent primordia and by driving the increased growth rate associated with organogenesis. As the organ grows, it depletes auxin from its vicinity by active auxin transport towards the organ, and this leads to auxin depletion in the area around the organs, where no new organs thus can be initiated. The combination of these inhibitory fields would only leave one site where auxin is most likely to accumulate, namely where the next primordium will emerge (Reinhardt *et al.*, 2003; Besnard *et al.*, 2011).

**Fig. 1.**
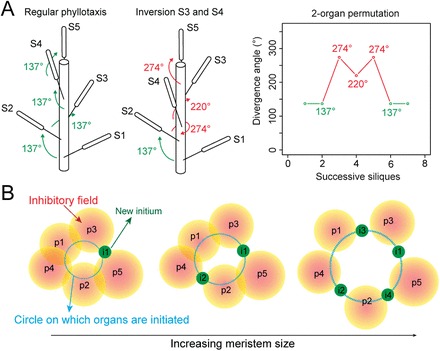
Model: how increasing meristem size can promote organ permutations. (A) Graphical definition of an organ permutation, based on silique positions on the stem (left panels): non-canonical divergence angles (red) are generated in the phyllotactic sequence (right panel). (B) Theoretical impact of a modulation of meristem size on the sequence of organ emergence in the SAM, assuming that organ inhibitory fields are not scaling to meristem size and that no change of phyllotactic mode occurs.

However, when analysing the phyllotactic sequences carefully, it appears that phyllotaxis is not as robust as the spirals observed in sunflower capitula, for instance, would suggest. In particular, while most divergence angles along the *Arabidopsis* stem are ~137°, other angles of ~50°, 80°, 220°, or 274° are often observed. Some of these aberrant angles were predicted (Couder, 1998), and recently shown, to reflect organ co-initiation at the SAM and subsequent permutations in the sequence of organs along the stem. For instance, the permutation in the sequence of two successive organs along the stem leads to a first angle of ~274° (2α) followed by an angle at ~220° (–α), followed by another angle at ~274° (Fig. 1A; Guedon *et al.*, 2013; Besnard *et al.*, 2014). Analysis of a mutant in the ARABIDOPSIS HISTIDINE PHOSPHOTRANSFER PROTEIN 6 (AHP6) demonstrated that this apparent noise is controlled genetically (Besnard *et al.*, 2014).

In addition to biochemical factors, geometrical factors also contribute to phyllotaxis. In particular, in a seminal study, the diversity of phyllotactic patterns observed in nature was reproduced by modulating a single geometrical factor (Γ), which only depends on the diameter of the ring on which new organs are generated (relating to meristem size) and on the diameter of organs (including their inhibitory field, i.e. a region around the organ where no new organ can emerge) (Douady and Couder, 1992, 1996*a*, b, *c*). In the footsteps of this work, a model recently predicted that the frequency of permutations on the stem, and thus the robustness of phyllotaxis, is also determined by meristem size, when assuming that organ inhibitory fields are not scaling to meristem size (Mirabet *et al.*, 2012; Fig. 1B). This prediction has not, however, been tested experimentally. Here morphometric tools were used to investigate this question in different growth conditions and genotypes, finally validating the model prediction.

## Materials and methods

### Plant lines and growth conditions

Seeds *of Arabidopsis thaliana*, ecotypes Wassilewskija (WS) and Colombia (Col-0), were provided by the Arabidopsis Biological Resource Centre (Ohio State University, Columbus, OH, USA). *Clasp-1* was kindly provided by G. Wasteneys and has been described previously (Ambrose *et al.*, 2007). Note that WS-4 has been shown to carry a mutation in the phytochrome D gene (Aukerman *et al.*, 1997). The *pAHP6::GFP* line has also been described previously (Besnard *et al.*, 2014).

### Observations of dissected meristems

To access the inflorescence meristem, flowers and floral buds (down to stage 3; Smyth *et al.*, 1990) were dissected out; the stem apices were then kept in an apex culture medium (Hamant *et al.*, 2014). Dissected meristems were imaged as previously described (Landrein *et al.*, 2013).

### Morphometry

To measure meristem size, the three smallest and successive stage 2 and 3 organs (as defined in Smyth *et al.*, 1990) were used to draw three meristem radii (labelled in yellow on the central panel in Supplementary Fig. S1B available at *JXB* online) and their intersection determined the geometrical centre of the SAM surface. Next, the length between the centre of the SAM and the extremity of the peripheral zone was measured on the next two successive stage 1 organs: two bars (labelled in blue on the right panel in Supplementary Fig. S1B) were drawn following the spatial pattern of phyllotaxis. The average of these two bars was then used to define the meristem size, as the mean radius of the meristem. To measure the plastochrone ratio R, the distance between the centre of the SAM and the external edge of successive primordia was measured (Supplementary Fig. S2A).

To assess the organ size in the meristem, projections of confocal *z*-stacks of meristems labelled with FM4-64 were used and the surface of each stage 2 and stage 3 primordium was measured by drawing a circle around each primordium using the free-hand tool of ImageJ and ensuring that the meristems were strictly oriented perpendicularly to the *z*-plane (Supplementary Fig. S1E at *JXB* online). The difference of size between successive primordia was calculated by ordering them following the phyllotactic spiral (Supplementary Fig. S1E).

Measurements of the divergence angles on the stem were performed as previously described (Landrein *et al.*, 2013).

### Statistical analysis

Statistical analyses were performed using either Excel or R softwares. On each graph with error bars, data are represented as ± the confidence interval (α=0.05%). Two-tailed Student tests were performed to compare means of independent biological replicates. Non-parametric Fisher tests were done to compare frequencies. Data were considered as statistically different when the *P*-value was inferior to the α=0.05% standard threshold.

## Results

### Meristem size is positively correlated to the number of organ permutations in the phyllotactic sequence

**Fig. 2.**
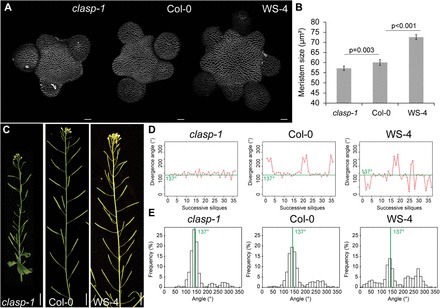
Meristem size and the robustness of phyllotaxis in *Arabidopsis*. (A) Representative shoot apical meristems in *clasp-1*, Col-0, and WS-4 labelled with FM4-64. Scale bars=20 μm. (B) Meristem size in *clasp-1* (*n*=24), Col-0 (*n*=28), and WS-4 (*n*=29), ***P<0.001 by two-tailed Student tests. (C) *clasp-1*, Col-0, and WS-4 inflorescence stems. Scale bars=1cm. (D) Divergence angles between the successive siliques in representative *clasp-1*, Col-0, and WS-4 sequences. (E) Distribution of divergence angle frequencies between successive siliques in *clasp-1* (619 angles, 17 plants), Col-0 (703 angles, 23 plants), and WS-4 (1046 angles, 25 plants) stems.

Three different genotypes with significant differences in meristem size were used (see the Materials and methods; Supplementary Fig. S1A, B at *JXB* online): meristems from plants of the WS-4 ecotype (*n*=29) were significantly larger than those of plants of the Col-0 ecotype (*n*=28; *P*<0.001, two-tailed Student test); meristems from plants of the Col-0 ecotype were themselves significantly larger than those of the *clasp-1* mutant, impaired in a previously characterized microtubule-associated protein (*n*=24, *P*=0.0031, two-tailed Student test, Fig. 2A, B; Ambrose *et al*.. 2007). This ranking also correlated with plant stature (Supplementary Fig. S1A at *JXB* online). Next it was investigated whether such differences in meristem size could be correlated to defects in the position of successive siliques along the stem (Fig. 2C). Using a previously described method (Peaucelle *et al.*, 2008), the divergence angles between successive siliques on the stem were measured in the three genotypes. Representative sequences of divergence angles along individual stems are presented in Fig. 2D. Qualitatively, the *clasp-1* mutant exhibited the most robust final phyllotactic pattern, while WS-4 seemed the most perturbed. The analysis of the divergence angle distribution confirmed this observation (Fig. 2E): the highest proportion of angles near 137° were obtained in *clasp-1* (619 angles, 17 plants) while the lowest was in the WS-4 background (1046 angles, 25 plants). Two peaks at ~220° (–α) and 274° (2α) were observed in all sequences; these were flatter in *clasp-1* and taller in WS-4. Interestingly, an additional peak at ~50° (–2α) and 80° (3α) was also observed in WS-4 only (Fig. 2E). Because these angle signatures have been related to organ permutations in *Arabidopsis* inflorescences (Guedon *et al.*, 2013; Besnard *et al.*, 2014) and in sunflower vegetative stems (Couder, 1998), it was next investigated whether these defects can be attributed to permutations in the temporal sequence of organ emergence.

Using a previously described combinatorial model with von Mises distribution representing divergence angle uncertainty (Refahi *et al.*, 2011; Guedon *et al.*, 2013), it was found that most of the aberrant angles can indeed be attributed to permutations between successive siliques (Table 1). The rate of permutations in *clasp-1* was ~9%, and this is significantly different from Col-0, which exhibited permutations for 14% of the siliques (*P*=0.003, comparison of frequencies, Table 1). In both genotypes, the vast majority of permutations were single permutations between two successive siliques (Table 1; Supplementary Fig. S1C, D at *JXB* online: 2α, –α angles) as previously observed in Col-0 (Guedon *et al.*, 2013; Besnard *et al.*, 2014). The number of permutations was increased in WS-4 (43%, statistically different from *clasp-1* and Col-0, *P*<0.001, comparison of frequencies) and the model also detected a significant number of permutations between three successive siliques (Table 1; Supplementary Fig. S1C, D) that generates additional angles such a –2α and 3α, consistent with the presence of numerous divergence angles at ~50° and to a lesser extent 80° in the distribution (Fig. 2E). Taken together, these findings show that meristem size is positively correlated to the number of permutations in the phyllotactic sequence.

**Table 1.** Analysis of the succession of divergence angles between successive siliques on the stem

	Short then long days			Long days only		
	*clasp-1*	Col-0	WS-4	*clasp-1*	Col-0	WS-4
Meristem size (radius in μm)	57.2	60.1	72.6	51.7	55.1	59.0
Number of angles	619	704	1046	667	1193	487
Number of sequences	17	29	25	19	34	15
Non-canonical angles (%)	14.8	23	60.5	11.2	5.7	16
Unexplained angles (%)	1.1	2.7	7.7	4.3	2.6	3.6
Number of two-organ permutations	28	49	154	14	13	22
Number of three-organ permutations	1	2	52	1	0	1
Permutated organs (%)	9.2	14	43.3	4.2	2.1	9.3
Mean angle α	138	136	136	128	136	141
Number of Lucas sequences	0	0	0	6	0	0

### Meristem size is negatively correlated to the plastochrone

**Fig. 3.**
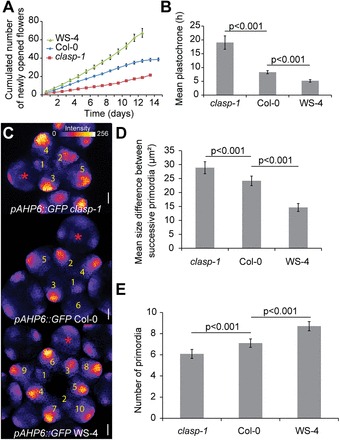
Plastochrone in *clasp-1*, Col-0, and WS-4. (A) Cumulated number of newly opened flowers in *clasp-1* (n=16), Col-0 (n=24), and WS-4 (n=17). (B) Mean plastochrone calculated from the first 10 d of flower production (two-tailed Student test). (C) Representative *clasp-1*, Col-0, and WS-4 meristems expressing the *pAHP6::GFP* marker of organ identity, using the ImageJ Fire lookup table. Scale bars=20 μm. (D) Mean size difference between successive primordia in *clasp-1*, 147 organs, 26 meristems; Col-0, 148 organs, 23 meristems; WS-4, 161 organs, 20 meristems, two-tailed Student tests. (E) Quantification of the number of GFP-positive stage 1 and stage 2 primordia in *clasp-1* (*n*=12 meristems), Col-0 (*n*=20 meristems), and WS-4 (*n*=17 meristems), two-tailed Student tests.

Theoretically, a larger meristem leads to a higher rate of organogenesis; that is, a reduced absolute plastochrone length (Fig. 1B; Douady and Couder, 1996*c*), assuming negligible changes in the inhibitory fields. If the age difference between successive primordia is reduced, this in turn would make permutations more likely (Mirabet *et al.*, 2012; Besnard *et al.*, 2014). To test whether the relationship between meristem size and number of permutations depends on the rate of organ emergence, the plastochrone was next measured by quantifying the number of newly opened flowers every day during 15 d in *clasp-1*, Col-0, and WS-4. Consistent with the meristem size ranking, the number of newly opened flowers each day was the lowest in *clasp-1* and the highest in WS-4 (Fig. 3A). Based on the first 10 d of these sequences, a plastochrone was measured of 19h in *clasp-1*, 8h for Col-0, and 5h for WS-4 (Fig. 3B; *clasp-1*, 16 plants; Col-0, 24 plants; WS-4, 17 plants; two-tailed Student test, *P*<0.001 for the comparisons Col-0/*clasp-1* and Col-0/WS-4).

To check whether this conclusion is also true at the meristem, the average size of each organ was next measured and the mean size difference between successive organs was calculated. Note that this measurement is relatively independent of the presence of permutations, as absolute values are reported. The largest difference in size between successive organs was observed in *clasp-1* and the smallest in WS-4 (Fig. 3D; *clasp-1*, 147 organs, 26 meristems; Col-0, 148 organs, 23 meristems; WS-4, 161 organs, 20 meristems; *P*<0.001, two-tailed Student test), consistent with the measured plastochrones. Because this measurement can be slightly biased by the observed shape of each primordium, the number of young organs at a given time point in the meristems was also counted using the transcriptional fusion *pAHP6::GFP*, as a marker of young primordium identity (Besnard *et al.*, 2014). To focus on the early steps of organ emergence only, all stage 1 and stage 2 primordia were included in the analysis, but older organs, notably organs at stage 3 in which the green fluorescent protein (GFP) signal appeared in the lateral sepals (labelled by a red asterisk in Fig. 3C, stages as defined by Smyth *et al.*, 1990), were excluded. This quantification was again consistent with the measured plastochrone: the average number of young organs was the highest in WS-4 and the lowest in *clasp-1* (Fig. 3E; *clasp-1*, *n*=12 meristems; Col-0, *n*=20 meristems; WS-4, *n*=17 meristems; *P*<0.001, two-tailed Student test for *clasp-1/*Col-0 and Col-0/WS-4). Note that as the *pAHP6::GFP* marker line is in the Col-0 background, the quantifications in WS-4 were performed on F_3_ plants from a segregation of the interecotype crossing and not on a pure WS-4 background. Based on the measurement of the organogenesis rate in the inflorescence, on the size difference between successive organs in the meristem, and on the average number of young primordia in meristems, it is thus concluded that meristem size is negatively correlated to the plastochrone. This in turn is consistent with the idea that increased noise in the phyllotactic sequence is caused by more frequent organ permutations when meristem size increases.

### Day length impacts meristem size and the robustness of phyllotaxis

At this stage, it cannot be excluded that the gradient observed in the different genotypes is linked to genetic regulation, independent of meristem size (for a link between CLASP and auxin distribution, see, for example, Ambrose *et al.*, 2013). To check for this possibility, different day length conditions were used to affect the size of the meristem and the same analysis as above was performed, within one genotype: plants were either grown in short days for 1 month after germination and then transferred to long days (the conditions used to obtain the above results) or only grown in long days from germination.

**Fig. 4.**
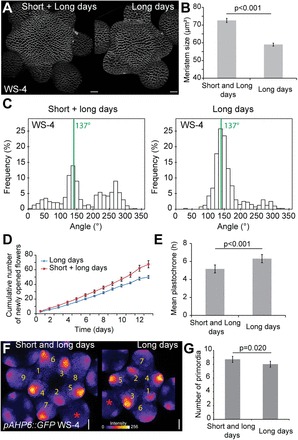
Impact of day length on the robustness of phyllotaxis in WS-4. (A) Representative WS-4 meristems labelled with FM4-64 and grown in two different conditions. Scale bars=20 μm. (B) Meristem size in plants grown in short and long days (*n*=29) or in long days only (*n*=15), two-tailed Student test. (C) Distribution of divergence angle frequencies between successive siliques in plants grown in short and long days (1046 angles, 25 plants) or in long days only (487 angles, 15 plants). (D) Cumulated number of newly opened flowers in short and long days (*n*=17 plants) or in long days (*n*=14 plants). (E) Mean plastochrone calculated from the first 10 d of flower production (two-tailed Student test). (F) Representative meristems from *pAHP6::GFP* lines grown in two different conditions. GFP signal intensity is represented using the ImageJ Fire lookup table. Scale bars=20 μm. (G) Number of GFP-positive stage 1 and 2 primordia in meristems from plants grown in two different conditions (*n*=17 meristems for short and long day condtion and *n*=16 for long day condition, two-tailed Student test).

First, plants from the WS-4 ecotype were analysed. Growth in long day conditions led to the formation of smaller meristems (*n*=15) in comparison with short then long day conditions (*n*=29 meristems; *P*<0.001, two-tailed Student test, Fig. 4A, B). Consistent with the previous results, the reduction of meristem size in WS-4 in long days was correlated with an increase in the frequency of 137° divergence angles in the phyllotactic sequences (Fig. 4C) and a reduction of the frequency of permutations observed on the stem (Table 1; short and long days, 1046 angles, 25 plants; long days only, 487 angles, 15 plants; *P*<0.001, comparison of frequencies). This reduction of meristem size was also correlated to an increase of the plastochrone, as assessed by measuring the number of newly opened flowers every day (Fig. 4D). The mean plastochrone, as estimated from the first 10 d of flower production, was 5.1h in short and long day conditions (*n*=17 plants) and 6.3h in long day conditions (*n*=14 plants; *P*<0.001, two-tailed Student test, Fig. 4E). This reduction of the plastochrone was also confirmed by measuring the number of young primordia in *pAHP6::GFP* meristems (Fig. 4F, G; *n*=17 meristems in short and long day conditions, *n*=16 meristems in long day conditions; *P*=0.020, two-tailed Student test).

**Fig. 5.**
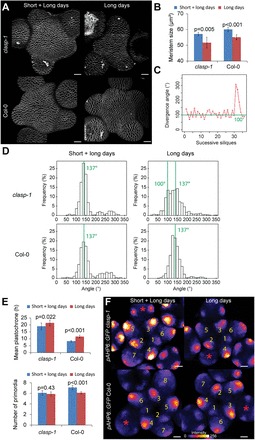
Impact of day length on the robustness of phyllotaxis in Col-0 and *clasp-1*. (A) Representative Col-0 and *clasp-1* meristems labelled with FM4-64 and grown in two different conditions. Scale bars=20 μm. (B) Meristem size in plants grown in short and long days (Col-0, *n*=28; *clasp-1*, *n*=24) or in long days only (Col-0, *n*=15; *clasp-1*, *n*=18; two-tailed Student tests). (C) Succession of ~100° divergence angles between the successive siliques in a *clasp-1* stem. (D) Distribution of divergence angle frequencies between successive siliques in plants grown in short and long days (Col-0, 703 angles, 26 plants; *clasp-1*, 619 angles, 17 plants) or in long days (Col-0, 1190 angles, 34 plants; *clasp-1*, 667 angles, 19 plants). (E) Top: mean plastochrone calculated from the first 10 d of flower production (two-tailed Student test) in short and long days (Col-0, *n*=24; *clasp-1*, *n*=16) or in long days only (Col-0, *n*=19; *clasp-1*, *n*=23; two-tailed Student tests). Bottom: number of GFP-positive stage 1 and 2 primordia in meristems from plants grown in short and long days (Col-0, *n*=20; *clasp-1*, *n*=12) or in long days only (Col-0, *n*=20; *clasp-1*, *n*=15; two-tailed Student tests). (F) Representative meristems from the *pAHP6::GFP* lines grown in the two different conditions. GFP signal intensity is represented using the ImageJ Fire lookup table. Scale bars=20 μm. Note that graphs in short then long days are reproduced from Fig. 5 for convenience.

A similar effect of day length conditions was observed on meristem size and on the number of silique permutations in both Col-0 and *clasp-1* (Fig. 5; Table 1). In the SAM, results in Col-0 were also consistent with previous findings (Fig. 5E, F). However, the quantification of the number of young primordia in *clasp-1* meristems did not reveal statistical differences between the two growth conditions (Fig. 5E). This exception might be explained by two hypotheses. First, the number of primordia is an integer, making it more difficult to detect changes in this number induced by small changes in plastochrone. Secondly, meristem size may reach a threshold beyond which the plastochrone reaches a plateau. Consistent with these hypotheses, the appearance of Lucas sequences (succession of ~100° divergence angles) could be detected for the first time in six phyllotactic sequences out of 19 in the *clasp-1* mutant in long day conditions, suggesting that the meristem could be small enough to shift to a new phyllotactic mode (Fig. 5C, D). Such behaviour was predicted in previous theoretical work, notably by modulating the factor Γ (Douady and Couder, 1996c). At this stage, this remains a hypothesis, as, to the authors’ knowledge, only an alteration of auxin signalling in multiple *plethora* mutants was shown to induce patterns of phyllotaxis other than Fibonacci in *Arabidopsis* meristems (Prasad *et al.*, 2011).

Finally, to confirm the results further, the plastochrone ratio (R) was measured in all these accessions and growth conditions. R is defined as the ratio of the distances of two consecutive primordia from the centre of the apex (Suplementary Fig. S2A at *JXB* online) and is an indirect way to quantify inhibitory fields between successive organs (Richards, 1948, 1951; Erickson and Meicenheimer, 1977; Lyndon, 1978; Meicenheimer, 1979). When pooling all the data together, a negative correlation between R and meristem size (Supplementary Fig. S2B), and thus a positive correlation between R and the robustness of phyllotaxis, was found. Note, however, that the small size of the *Arabidopsis* meristem led to a high level of noise in measurement of the organ to meristem centre distances, and, therefore, the trend was not significant for the smallest meristems (Supplementary Fig. S2C).

## Discussion

Taken together, the data support a scenario in which an enlarged meristem size promotes a shorter plastochrone, leading to an increase in the frequency of organ permutations and less robust phyllotactic patterns, thus validating previous model predictions (Mirabet *et al.*, 2012). The negative correlation between meristem size and plastochrone found in this study is actually consistent with previous observations in various flowering plants, where an increase in meristem size during the transition from vegetative to reproductive growth is correlated with an acceleration of organ production (Erickson and Meicenheimer, 1977; Lyndon, 1978; Meicenheimer, 1979; Rutishauser, 1998). Although this was not apparent in the present experiments, the possibility that the plastochrone contributes to phyllotactic robustness independently of meristem size cannot be excluded.

Interestingly, the present study also shows that, while the robustness of phyllotaxis depends on the genotype and is not maximal in the wild type, environmental growth conditions can interfere and even partially rescue phyllotactic defects, by affecting meristem size. Incidentally, this study also suggests that in the *Arabidopsis* meristem, inhibitory fields are not scaling to meristem size, as the variations in robustness suggest that inhibitory fields remain relatively constant while meristem size is modulated. This is probably not true in all plants: for instance, while screening SAMs of several flowering plants, Rutishauser (1998) concluded that Fibonacci spiral patterns tend to shift to Lucas patterns (and other accessory spiral patterns) more frequently when the meristems are large enough as compared with the primordia nearby (i.e. when R is closer to 1). This is in contradiction to the findings of the present study, and could suggest that inhibitory fields are not always scaling to meristem size. As more data become available, the comparison of phyllotaxis, and its robustness, across species could thus help better understand the link between the establishment of patterns and its regulation.

Phyllotaxis has been studied from various angles, although, in the past decades, the main focus has been on the molecular mechanisms behind organ emergence (e.g. Fleming *et al.*, 1997; Reinhardt *et al*., 2000, 2003; Heisler *et al.*, 2005; Peaucelle *et al*., 2011*a*, *b*; Nakayama *et al.*, 2012; Braybrook and Peaucelle, 2013) and how it can explain the production of regular patterns (e.g. Kuhlemeier and Reinhardt, 2001; Jonsson *et al.*, 2006; Smith *et al.*, 2006; Stoma *et al.*, 2008; Bayer *et al.*, 2009; Heisler *et al.*, 2010). The alterations in phyllotactic patterns have received less attention, although growth has clearly been shown to play a key role in this respect. For instance, ectopic expression of the *CUP SHAPED COTYLEDON* genes affects the variability of internode length, leading to major defects in the final phyllotactic sequence (Peaucelle *et al.*, 2007; Sieber *et al.*, 2007). Similarly, induction of right-hand stem torsion in internodes in the *cellulose synthase interactive protein 1* and *spiral 2* mutants leads to a bimodal phyllotactic pattern (Landrein *et al.*, 2013). In both examples, the phyllotactic pattern in the SAM is not affected and thus all defects are strictly related to post-meristematic growth. Growth defects can also occur earlier, such as within the meristem, and also lead to phyllotactic defects. In particular, mutation in the meristem-expressed cytokinin transducer gene *ABPHYL1* leads to a shift in the phyllotactic mode, from alternate to decussate, in maize (Giulini *et al.*, 2004). In *Arabidopsis*, two successive organs can emerge at roughly the same time in the meristem, leading to permutations in the final sequence. This effect is amplified in the cytokinin signalling mutant *ahp6*, thus demonstrating a role for cytokinin signalling in the regulation of the tempo of organogenesis at the SAM (Besnard *et al.*, 2014). In the present study, this conclusion was generalized by adding another parameter, meristem size, and it was demonstrated that it is sufficient to explain the measured variability in phyllotaxis in the wild type and in different growth conditions.

This work supports an emerging concept in developmental biology: the role of time in the variability of patterns. For instance, variability in the timing of gene expression has attracted more attention recently as it appears to have a much more important role in development than simply adding white noise. For instance, it has been theoretically shown that time delays due to gene expression dynamics (e.g. transcription) can have a dramatic impact on morphogen-based patterning (Seirin Lee *et al.*, 2010), notably less robustness with respect to noise. Although at a much larger scale, the present study follows a relatively similar idea: by increasing the variability in the timing of organ emergence, the final spatial phyllotactic pattern becomes less robust, even though the initial pattern is unaffected. In other words, robustness in patterning depends on time-dependent noise at all scales.

Taking a step back, the focus on meristem geometry in the present study recalls the very first models behind phyllotaxis, where each new primordium was proposed to appear following steric constraints, namely in the largest space available between pre-existing primordia within the SAM (Hofmeister, 1868). Although wrong mechanistically *sensu stricto*, this rule helped to define the concepts of chemical-based inhibitory fields. As it is shown here that meristem geometry impacts the temporal sequence of organ initiation, it now appears that the Hofmeister rule is rather true if the focus is not on the angles at which each new organ is initiated, but instead on the regularity of the phyllotactic pattern (Fig. 1B).

## Supplementary data

Supplementary data are available at *JXB* online.

Figure S1. Morphometry.

Figure S2. The plastochrone ratio R and meristem size.

Supplementary Data
