# Supplementary material for: Meristem size contributes to the robustness of phyllotaxis in Arabidopsis
Source: J Exp Bot. 2014 Dec 11;66(5):1317–24. doi: 10.1093/jxb/eru482 (PMC4339594; doi:10.1093/jxb/eru482)
Supplement: Supplementary Data [file supp_66_5_1317__index.html]

Meristem size contributes to the robustness of phyllotaxis in Arabidopsis — Meristem size contributes to the robustness of phyllotaxis in Arabidopsis — Supplementary Data 

# Meristem size contributes to the robustness of phyllotaxis in *Arabidopsis*

## Supplementary Data

Data files

**Files in this Data Supplement:**

- Supplementary Data - Supplementary Data
