# Supplementary material for: Meristem size contributes to the robustness of phyllotaxis in Arabidopsis
Source: J Exp Bot. 2014 Dec 11;66(5):1317–24. doi: 10.1093/jxb/eru482 (PMC4339594; doi:10.1093/jxb/eru482)
Supplement: Supplementary Data [file supp_eru482_jexbot133587_file001.pdf]

Meristem size contributes to the robustness of phyllotaxis in *Arabidopsis*

Benoit Landrein[1,2], Yassin Refahi[1,3], Fabrice Besnard[1,4], Nathan Hervieux[1,2], Vincent Mirabet[1,2], Arezki Boudaoud[1,2,5], Teva Vernoux[1], Olivier Hamant[1,2,*]

1. Laboratoire de Reproduction de développement des plantes, INRA, CNRS, ENS Lyon, UCB Lyon 1, Université de Lyon, 46 Allée d'Italie, 69364 Lyon, Cedex 07, France

2. Laboratoire Joliot-Curie, Laboratoire de Physique, CNRS, ENS Lyon, UCB Lyon 1, Université de Lyon, 46 Allée d'Italie, 69364 Lyon Cedex 07, France

3. Sainsbury Laboratory, University of Cambridge, Cambridge, United Kingdom

4. Present address: IBENS, ENS, 75005 Paris, France

5. Institut Universitaire de France, 103, boulevard Saint-Michel, 75005 Paris, France

* Correspondence to: olivier.hamant@ens-lyon.fr, Tel: +33 (0)4 72 72 88 75 fax: +33 (0)4 72 72 86 00, Laboratoire de Reproduction de développement des plantes, INRA, CNRS, ENS Lyon, UCB Lyon 1, Université de Lyon, 46 Allée d'Italie, 69364 Lyon, Cedex 07, France

Running head: Meristem size and phyllotaxis

5 figures, 1 table, 2 supplementary figures

Word count: 4200 words

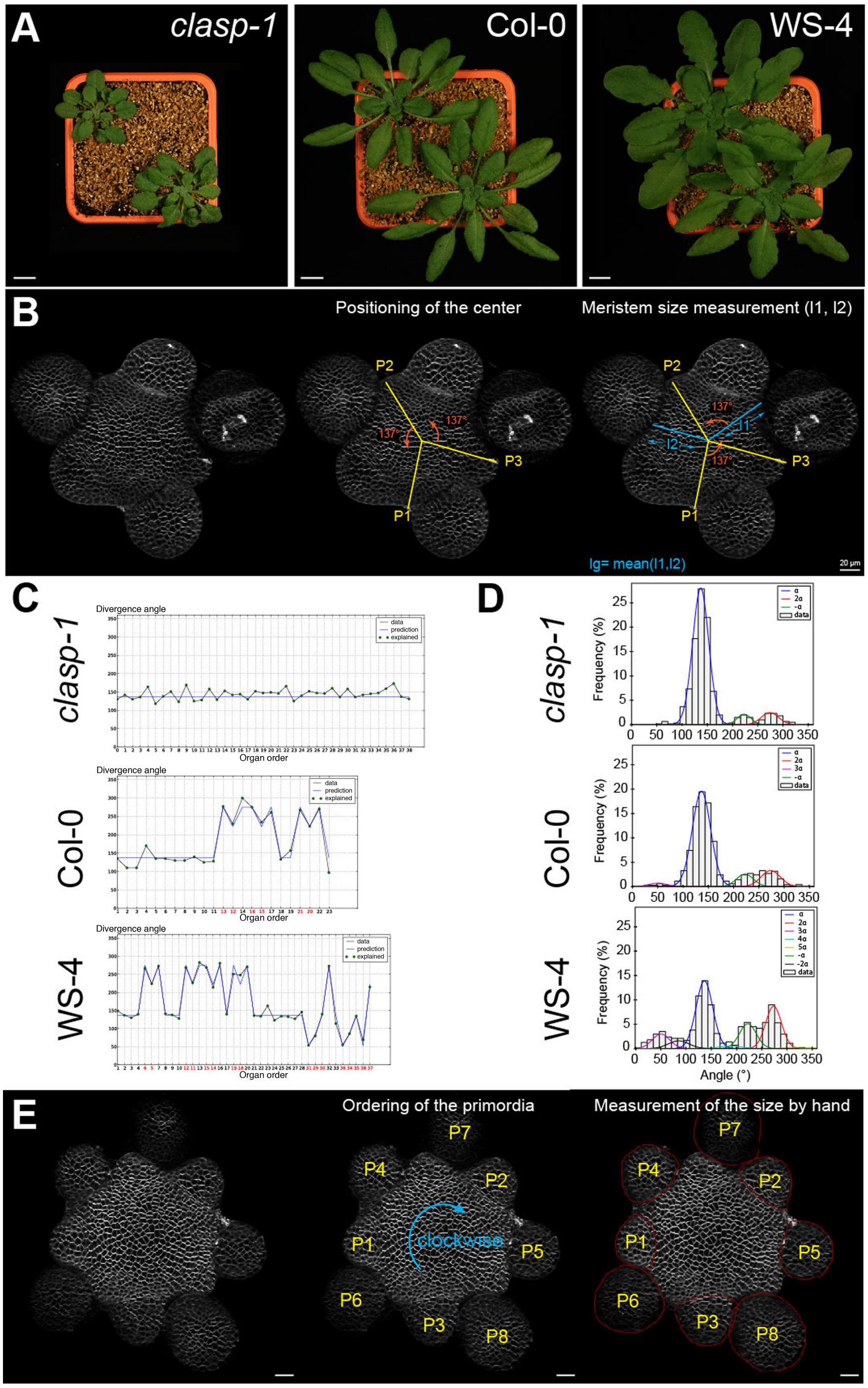

Figure S1. Morphometry

A. Representative *clasp-1*, Col-0 and WS-4 plants grown one month in short days and one week in long days. Scale bar, 1 cm. **B.** Meristem size measurement (see methods). **C.** Examples of phyllotactic sequences (different from fig. 3D) where the permutations in the organ sequence (in red) have been explained using the combinatorial model (Refahi et al., 2011). **D.** Distribution of divergence angle frequencies with a fit from the combinatorial model (Refahi et al., 2011). **E.** Organ size measurement and ordering (see methods).

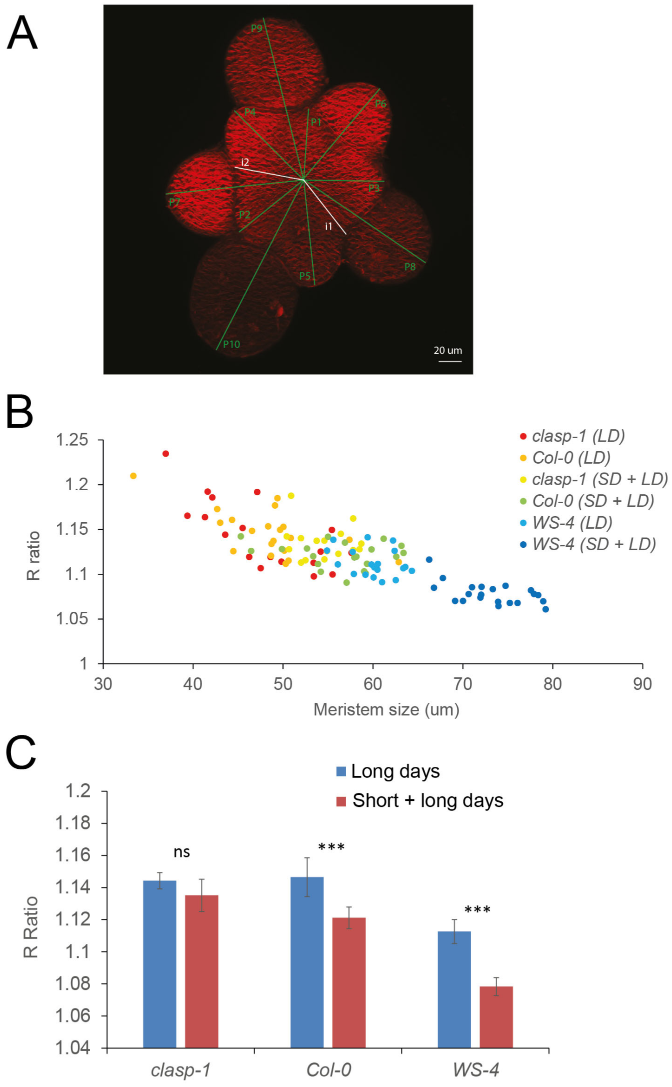

Figure S2. The plastochrone ratio R and meristem size

A. The distances between the center of the SAM and the external edge of two successive primordia were measured (using the same original images as for the rest of the article) and the corresponding ratio R was defined as the plastochrone ratio. **B.** Scatter plot compiling all measurements showing a negative correlation between R and meristem size in WS-4, Col-0, *clasp-1* in short then long days or in long days only. Each dot represents the average meristem size and plastochrone ratio for a given meristem. **C.** The impact of day length on R was statistically significant for WS-4 and Col-0 ($p<0.001$) but not significant for *clasp-1*. When comparing different accessions within a given day length condition, the difference in R between WS-4 and Col-0 or between WS-4 and *clasp-1* was statistically different ($p<0.001$) both in short then long days and long days only. The difference in R between Col-0 and *clasp-1* was still statistically different in short then long days ($p<0.05$) but became not significant in long days only.
